# Social Disparities in Nitrate-Contaminated Drinking Water in California’s San Joaquin Valley

**DOI:** 10.1289/ehp.1002878

**Published:** 2011-06-03

**Authors:** Carolina Balazs, Rachel Morello-Frosch, Alan Hubbard, Isha Ray

**Affiliations:** 1 Energy and Resources Group,; 2 School of Public Health, and; 3 Environmental Science Policy and Management, University of California, Berkeley, California, USA

**Keywords:** California, drinking water, environmental justice, nitrate, public health, Safe Drinking Water Act, social disparities, water systems

## Abstract

Background: Research on drinking water in the United States has rarely examined disproportionate exposures to contaminants faced by low-income and minority communities. This study analyzes the relationship between nitrate concentrations in community water systems (CWSs) and the racial/ethnic and socioeconomic characteristics of customers.

Objectives: We hypothesized that CWSs in California’s San Joaquin Valley that serve a higher proportion of minority or residents of lower socioeconomic status have higher nitrate levels and that these disparities are greater among smaller drinking water systems.

Methods: We used water quality monitoring data sets (1999–2001) to estimate nitrate levels in CWSs, and source location and census block group data to estimate customer demographics. Our linear regression model included 327 CWSs and reported robust standard errors clustered at the CWS level. Our adjusted model controlled for demographics and water system characteristics and stratified by CWS size.

Results: Percent Latino was associated with a 0.04-mg nitrate-ion (NO_3_)/L increase in a CWS’s estimated NO_3_ concentration [95% confidence interval (CI), –0.08 to 0.16], and rate of home ownership was associated with a 0.16-mg NO_3_/L decrease (95% CI, –0.32 to 0.002). Among smaller systems, the percentage of Latinos and of homeownership was associated with an estimated increase of 0.44 mg NO_3_/L (95% CI, 0.03–0.84) and a decrease of 0.15 mg NO_3_/L (95% CI, –0.64 to 0.33), respectively.

Conclusions: Our findings suggest that in smaller water systems, CWSs serving larger percentages of Latinos and renters receive drinking water with higher nitrate levels. This suggests an environmental inequity in drinking water quality.

An array of drinking water–related problems still exists in the United States, despite a history of investment in sophisticated water infrastructure and the existence of federal laws such as the Clean Water Act of 1972 and Safe Drinking Water Act of 1974 (SDWA) that regulate source contamination and protect the public’s health. These problems include increasing source contamination (Dubrovsky et al. 2010), exposure to chemical and microbial contaminants, poor implementation of water laws (Burke 2009; Duhigg 2009), and degrading infrastructure (Levin et al. 2002). Rural areas often face the largest burden, as aquifers are contaminated by intensive agriculture and livestock production (Dubrovsky et al. 1998). Some rural unincorporated areas, such as some communities along the U.S.–Mexico border, lack access to adequate infrastructure, service provision, and clean water (Olmstead 2004; Pilley et al. 2009).

Despite these problems, there is a paucity of studies that examine social disparities in exposure to unsafe water. A literature review in the 1990s (Calderon et al. 1993) recommended that more quantitative analyses examine whether vulnerable populations, including people of color and the poor, are disproportionately affected by drinking water contamination. Since then, a handful of studies have addressed different aspects of this issue. In San Joaquin County, California, one study found a weak but significant relationship between areas with higher poverty and greater proportions of minorities and poor drinking water quality (Byrne 2003). Research in the Navajo Nation found bacteriological and chemical contamination in unregulated drinking water sources (Murphy et al. 2009). In Arizona, researchers examined whether public water systems serving higher fractions of minority or low-socioeconomic-status (SES) residents were more likely to exceed the arsenic maximum contaminant level (MCL) than were those serving higher fractions of whites or high-SES residents. They found a positive association between the percentage of Latino residents and the likelihood of exceeding the MCL of arsenic. However, they concluded that environmental justice concerns were unwarranted for Latinos, because there was no difference between the percentages of Latinos who were served by water systems with and without violations. (Cory and Rahman 2009). In New Mexico, preliminary research documented high arsenic levels in drinking water sources that provided water to predominantly Latino border communities known as “colonias” (Pilley et al. 2009).

Our research addresses several methodological limitations of previous studies, particularly regarding appropriate unit of analysis, characterization of exposure, and scale. For example, Byrne (2003) estimated average trichloroethylene levels and MCL exceedances in drinking water systems and characterized exposure as a continuous measure across San Joaquin County; the community level, however, is more appropriate when considering community-level exposure. Cory and Rahman (2009) characterized the association between percent minority and a binary outcome of arsenic, rather than a continuous measure of arsenic levels, and they did not explore this association among smaller systems where they noted that most arsenic violations occurred.

Our study used the community as the unit of analysis to examine the relationship between nitrate concentration in community water systems (CWSs) and social factors. CWSs are public water systems that serve water year-round to at least 25 people or have > 15 service connections [U.S. Environmental Protection Agency (EPA) 2010b]. We characterized potential exposure to nitrate because it is one of the most common contaminants found in groundwater (Harter 2009; Spalding and Exner 1993) yet has received little attention regarding social disparities in exposure.

Nitrate in drinking water is associated with methemoglobinemia (i.e., “blue baby syndrome”) in infants (Fan and Steinberg 1996; U.S. EPA 2010a), although other risk factors include enteric infections (Charamandari et al. 2001; Hanukoglu and Danon 1996) and foods high in nitrates (Sanchez-Echaniz et al. 2001). Epidemiologic data also suggest an association between nitrate levels in drinking water and reproductive toxicity, developmental effects, and various cancers (Fan and Steinberg 1996; Ward et al. 2005, 2010), although the consistency of these associations varies. To protect against methemoglobinemia, the SDWA has established an MCL of 45 mg/L as nitrate ion (NO_3_) or 10 mg/L as nitrate-nitrogen in drinking water (U.S. EPA 2010a).

California’s San Joaquin Valley is an important site for examining potential disparities in exposure to nitrate. With its intensive irrigated agriculture, the valley has two of the most contaminated aquifers in the nation and some of the highest nitrate levels in the country (Dubrovsky et al. 1998, 2010). Because nearly 95% of the valley’s population relies on groundwater for drinking [Permits, Inspections, Compliance, Monitoring and Evaluation (PICME) [California Department of Public Health (CDPH) 2008a], groundwater contamination is a particular health risk. This risk is compounded by the fact that with high costs of mitigation, few systems actually treat for nitrate. The San Joaquin Valley also has some of the highest rates of poverty and minority populations—particularly Latinos—in the state (U.S. Census Bureau 2007). These communities are economically and socially disadvantaged, making it harder for them to afford mitigation or to address related health consequences of nitrate contamination. The continued use of nitrogen-based fertilizers (Dubrovsky et al. 2010; Ruddy et al. 2006) and the increasing demand for groundwater (Glover 2010) further highlight the importance of this contaminant, because exposure may become increasingly widespread.

Given this context, we used water quality monitoring data from the CDPH to analyze the association between racial/ethnic and SES characteristics of people served by CWSs and nitrate levels of these systems in the San Joaquin Valley. With few exceptions (Byrne 2003), there has been limited use of CDPH monitoring data to examine whether certain groups are disproportionately affected by exposure to drinking water contaminants. Similarly, despite an acknowledgment of the burden faced by small systems (Committee on Small Water Systems 1997), few studies have explored associated social disparities.

We hypothesized that CWSs serving a higher percentage of minority or lower-SES residents have higher nitrate levels and that these disparities are likely to be greater among smaller drinking water systems. Disparities in nitrate exposures, if they exist, could signal a potential environmental injustice. This analysis expands the emerging literature on drinking water quality and social disparities in the United States and informs national- and state-level policy on the needs of underresourced water systems.

## Materials and Methods

Our units of observation were CWSs in California’s San Joaquin Valley. We used three measures to test our study’s hypotheses: *a*) estimated average nitrate concentrations for each CWS to describe average water quality served to customers; *b*) population potentially exposed (PEP) to three nitrate levels to estimate the population affected by nitrate contamination; and *c*) nitrate concentrations at points of entry into each CWS’s distribution system to assess the relationship between demographic characteristics of customers and CWS nitrate levels. The first two measures were used in a series of descriptive statistical analyses. The third measure was used as the outcome variable in linear regression models that estimated the relationships between race/ethnicity and SES and a system’s nitrate concentration.

**Figure 1:**
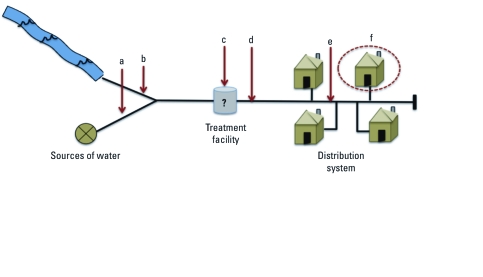
Schematic of a community water system (CWS) indicating the location of point-of-entry sources and the use of average nitrate concentration in the distribution system as a proxy for tap water quality. Water entering the distribution system may flow from a groundwater well (point a) or from a surface water source (i.e., stream; point b). Water may then be treated (point c; different treatment techniques may be used, depending on the contaminant of interest and original source). Water then enters the distribution system at points of entry (point d). In this example, nitrate samples would be used from point d, because points a and b flow into the same point of entry. Average nitrate level at point d is used to represent average water quality in the distribution system (point e). Nitrate levels in the distribution system are a proxy for tap water quality (point f). If points a and b are separate points of entry (i.e., do not flow into a shared point d), nitrate sample points would be used from each source separately. Constant and equal flows are assumed.

*Sample selection and time period.* We included CWSs that were active in the San Joaquin Valley between 1999 and 2001, had at least one point-of-entry source with a nitrate sample reported for this period, and had any source (i.e., point of entry or not) with geographic coordinate data available to estimate CWS demographics. Point-of-entry sources can be defined as sources of supply (e.g., well with no treatment or effluent from a well/surface water plant) that directly enter into the distribution system (Figure 1). We used nitrate-sampling data from 1999 to 2001 and demographic data from the 2000 Census. The sampling period represents one full compliance period under the SDWA [California Code of Regulations (CCR) 2008, §64400.25]. Of the 873 CWSs that were active during 1999–2001, 711 had sources with geographic coordinates. Of these, 327 (37%) had nitrate water quality sampling data and were included in our final sample.

*Average nitrate concentration for CWSs.* To estimate nitrate concentration for each CWS, we selected two types of point-of-entry sources for inclusion (Figure 1): *a*) sources, such as well fields or surface water plants, that were in active use and had no treatment or that were treated for contaminants other than nitrate, and *b*) treatment plants in active use that potentially treated for nitrate. In both cases, we included only sources that were last in line to enter the distribution system (i.e., did not flow to another source before entering), to avoid double counting of nitrate levels. We used the CDPH’s Permits, Inspections, Compliance, Monitoring and Enforcement database (CDPH 2008a) to identify source types, their location in relation to the distribution system, and their possible treatment techniques. If a plant had a treatment technique commonly used for nitrate (e.g., reverse osmosis), we assumed that it treated for nitrate.

We then used nitrate-sampling data for these sources from the CDPH (2008b) Water Quality Monitoring database to determine nitrate concentration at points of entry. This served as a proxy for water quality in each CWS’s distribution system and for tap water quality. Nitrate levels are unlikely to change from these entry points to the tap (unless systems chloraminate, which those in the valley do not) (Haberman R, personal communication). CWSs using groundwater are required to sample each source for nitrate annually (unless a single sample or average of two samples exceeds the MCL, in which case the system must sample quarterly); CWSs using surface water must sample quarterly (CCR 2008, §64432.1). In practice, however, systems often fail to sample regularly (Haberman R, personal communication). If a nitrate sample was below the detection limit of 2 mg NO_3_/L (CCR 2008, §64432), we took the square root of the value as a proxy for that sample’s nitrate level (Lubin et al. 2004). We did not have flow measurements for the individual sources that contributed water to each CWS’s distribution system. Therefore, we could not determine a flow-weighted measure of distribution water quality for each CWS based on the nitrate level measured in samples from each contributing source. Instead, we assumed that each point-of-entry source contributed independently and equally to a CWS’s distribution system, and that each source contributed a constant amount to the system, regardless of season.

Finally, we determined the average nitrate level for each point-of-entry source and averaged the resulting values across all sources to estimate an average systemwide nitrate level. The systemwide average was then used to categorize each CWS’s average nitrate concentration as *a*) low, defined as < MCL/2 (< 22.5 mg NO_3_/L); *b*) medium (22.5–44.9 mg NO_3_/L); and *c*) high (≥ 45 mg NO_3_/L, the MCL for nitrate). These categories correspond to those used to assess source-level nitrate concentrations for regulatory purposes (CCR 2008, §64432.1). Besides the high category, the medium category is important to consider because research suggests that exposure to nitrate in drinking water at half the MCL can cause adverse health effects among susceptible subpopulations (DeRoos et al. 2003). In addition to calculating average nitrate levels, we used nitrate MCL violation data from PICME to verify whether CWSs with high nitrates did in fact receive violations, and to run a sensitivity analysis on PEP data.

*Potentially exposed population.* Using a method by Storm (1994), we computed the potentially exposed population (PEP) by apportioning the total population served by each CWS into three exposure categories based on the proportion of sources for that CWS with average nitrate levels that were low, medium, or high, as defined above. The population in each category was then summed across all CWS to estimate the total population potentially exposed to the three nitrate levels. The approach to calculate the PEP for the high-nitrate category is summarized by the following equation:


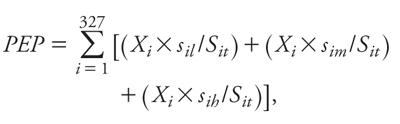
[1]

where *X_i_* is the total population served in CWS *i*; *s_ih_* is the number of sources for CWS *i* with average nitrate concentrations classified as high (*h*); and *S_it_* is the total number of point-of-entry sources for CWS *i*. To calculate the PEP for the low (*l*) or medium (*m*) nitrate categories, we replaced *s_ih_* with *s_il_* or *s_im_*, respectively. We used PICME 2008 data on the number of people served by each CWS to calculate the population size in each exposure category during 1999 to 2001. If the number of customers served by a CWS was not available from the PICME data, we used information from the CDPH Water Quality Monitoring database. To estimate population counts of potentially exposed individuals according to demographic characteristics (e.g., race/ethnicity) we multiplied the potentially exposed population in each nitrate category for each CWS by the estimated proportion of customers in each demographic subgroup for the CWS, and then summed these values across all CWS for each nitrate category. Because home ownership is based on housing units rather than population count, we did not derive a count of housing units.

*Statistical analysis of nitrate levels and CWS characteristics.* We used a linear regression model to analyze the relationship between CWS demographics and nitrate levels. We fitted a model selected *a priori* that controlled for known or hypothesized system-level confounders. We originally used a mixed model approach to account for clustering (Laird and Ware 1982). However, diagnostics of the mixed model indicated a very nonnormal distribution of residuals. Therefore, we used an approach that provided inference that was robust under laxer modeling assumptions. To derive the inference (i.e., standard errors), we clustered outcomes at the water system level (i.e., point-of-entry nitrate concentrations measured on a given day for a given source). Thus, our final model reported sandwich-type robust standard errors (Huber 1967) that allow for arbitrary correlation, including correlation within or across sources in a CWS.

Our outcome variable, *Y_ijk_*, is nitrate concentration for the *i*th water system, the *j*th source in system *i*, on day *k* (since 1 January 1999). Although nitrate samples from individual sources are our outcome measurements, the CWS is the primary unit of analysis, consistent with average nitrate level calculations discussed above. Our final model did not reweight CWSs with more samples (as the mixed model might have, depending on the implied estimated correlation structure), because we wanted CWS to contribute measurements based on a proxy of the number of people served. Thus, systems with more measurements contributed more to the estimates. We addressed this assumption by stratifying by system size, to see if smaller CWSs (with fewer samples) had a different effect than did larger CWSs. Because differences between the estimates of the mixed model and the linear model were small, this comparison provided evidence that the nonweighted approach of our final model was reasonable.

Key independent variables were the percentage of Latino and non-Latino people of color served by CWSs (referent category was non-Latino whites) and percent home ownership in the area served by a CWS. Latinos were analyzed separately because they are the largest ethnic group in the valley (40%; U.S. Census Bureau 2007). SES was represented by home ownership rate, which is a proxy for wealth and political representation (Krieger et al. 1997; Morello-Frosch et al. 2001; Oliver and Shapiro 1997). Because of our focus on CWS-level exposures, these variables were measured at the CWS level. We assumed these remained constant for all 3 years.

Race/ethnicity and home ownership data were derived from the 2000 U.S. Census (U.S. Census Bureau 2000). Because CWS service areas do not follow census boundaries, we used two spatial approaches in geographic information systems to estimate demographic variables for each CWS. We first compared an aerially weighted approach using digitized CWS boundaries in two pilot counties (Tulare and Fresno) with a second approach joining spatial coordinates from CDPH data for all sources (well fields, surface water intakes, and treatment plants) to census block groups. Based on spatial and goodness-of-fit comparisons, we concluded that it was reasonable to use the latter approach [for details on the aerially weighted approach and the comparison between the two methods, see Supplemental Material (http://dx.doi.org/10.1289/ehp.1002878)]. In brief, for each CWS, we estimated a population-based average of each variable across all block groups that included sources for the CWS. For example, if a CWS had two sources in two census block groups, we determined the population-weighted average of the variable across both census block groups and used that value to derive a percent estimate of demographic groups (e.g., 50% Latino) served by each CWS.

We controlled for other water system characteristics that could be potential confounders: source of water (groundwater or groundwater plus surface water vs. surface water alone), whether the system served a city (i.e., incorporated) or an unincorporated area, ownership structure of the system [publicly vs. privately owned and not regulated by the Public Utility Commission (PUC), with privately owned PUC-regulated systems as the referent category], system location (agricultural valley floor or not), season (summer/fall or winter/spring), year of sampling (2000 or 2001, with 1999 as referent category), and number of service connections (< 200 or ≥ 200 connections). CWSs with < 200 connections are generally considered “small” (CCR 2008, §64432.1). We determined ownership structure by combining data in PICME with data from the PUC’s list of regulated systems. We obtained all other characteristics from PICME. With the exception of year and season, which were measured at the source level, all covariates were measured at the water system level.

In addition to models including all CWSs, we stratified by system size to assess whether demographic effects on water quality might be stronger among smaller systems and to test the hypothesis that scale alone explains water quality. We also used our final model to estimate the amount of nitrate contamination attributable to the proportion of the population that is Latino. We did so by using the final model to predict expected values for each observation if percent Latino equaled zero, as described in Greenland and Drescher (1993). All statistical analyses were conducted using Stata (version 10; StataCorp LP, College Station, TX). We used Stata’s cluster command (clustering at the CWS level) to derive robust SEs.

## Results

*Descriptive statistics.* The 327 systems in our sample served approximately 2.95 million people, or 96% of the San Joaquin Valley population served by CWSs (Table 1). The distribution of average system-level nitrate concentrations is right-skewed and ranges from 0 to 150 mg NO_3_/L. This distribution and range are similar for average source-level nitrate concentrations and for individual sampling points (data not shown). The mean proportion of Latinos served across these CWSs was 32%, with an interquartile range (IQR) of 10–50%. The mean proportion of homeownership was 70%, with an IQR of 60–82%. Compared with all the CWSs in the valley active from 1999 to 2001, our study sample underrepresented small CWSs that have < 200 connections (49% vs. 73%; Table 1). The number of samples per source in systems with < 200 connections ranged from 1 to 110 (mean, 3.2), compared with a range of 1–133 (mean, 4.5) for systems with ≥ 200 connections. Six percent of samples had concentrations below the detection limit.

**Table 1:** CWSs included in study sample compared with all active CWSs, San Joaquin Valley, California, 1999–2001.

Variable of interest	Active CWSs with source location (*n* = 327)	CWSs in study (*n* = 327)	CWSs with < 200 connections (*n* = 160)	CWSs with ≥ 200 connections (*n* = 167)
Total population		3,047,822*a*		2,948,346		27,165		2,921,181
Latino population (%)		34		39		29		40
White population (%)		58		47		64		47
Population above poverty level*b* (%)		57		57		59		57
Population served (mean/median)		4,206/150		9,016/565		170/100		17,492/430
Incorporated*c* (%)		9		18		2		34
< 200 connections (*n*)		73		49		100		0
Only groundwater*d* (%)		89		90		97		84
Groundwater and surface water*d* (%)		5		8		3		13
**a**Approximately 71,418 people were served by CWSs whose sources did not have geographic coordinates, and 80 CWSs had no population estimates available; including these sources, the estimate of “true” population served by active CWSs was at least 3,119,240. **b**Above 200% of the poverty level. **c**A CWS that serves a city that is a legally recognized municipal corporation with a charter from the state and governing officials that is incorporated, as opposed to a water system that serves an unincorporated area. **d**Reference group, surface water only.								

**Figure 2:**
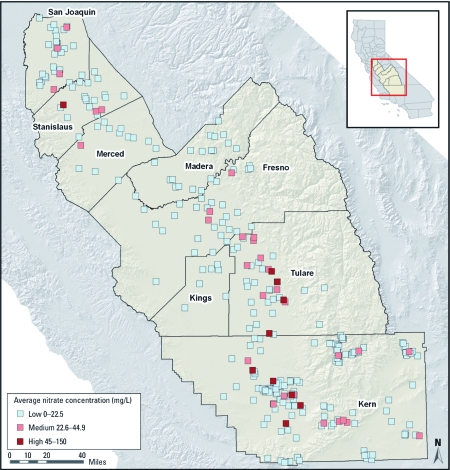
Average nitrate concentration of CWSs in California’s San Joaquin Valley, 1999–2001 (*n* = 327). Estimates are based on an average of each point-of-entry source’s average concentration. Data are from CDPH (2008) Water Quality Monitoring and PICME databases (CDPH 2008a, 2008b). Approximate locations of CWSs are depicted, but not true boundaries. Because of close proximity of some CWSs, some CWSs not fully visible.

Overall, 3% (*n* = 10) of all CWSs in our sample had high average nitrate concentrations (above the MCL of 45 mg NO_3_/L) for at least some part of the study period, 10% (*n* = 33) had medium average concentrations (MCL/2 to MCL), and 87% (*n* = 284) had low average concentrations (< MCL/2; Figure 2). Of the 10 CWSs with an average nitrate concentration > MCL, 9 had < 200 connections and 8 had only one or two sources. All but 1 of these 10 CWSs received at least one MCL violation during the study period, and 14 CWSs in our sample (serving ~ 92,268 people) received at least one MCL violation [PICME (CDPH 2008a); see also Supplemental Material, Table 1 (http://dx.doi.org/ 10.1289/ehp.1002878)].

**Figure 3:**
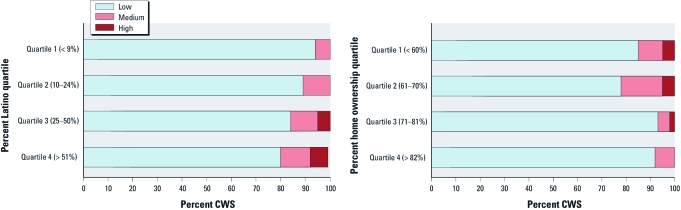
Percentage of CWSs with low, medium, and high average nitrate concentrations, by quartiles of percent Latino and home ownership. Average system-level nitrate concentration is derived from the average of each source’s average nitrate concentration at point of entry. Low is < one half the MCL of 45 mg NO_3_/L. Medium is one half the MCL up to the MCL (22.5 mg/L to 44.9 mg/L NO_3_). High is ≥ to the MCL.

CWSs that served higher fractions of Latinos and lower fractions of homeowners (i.e., more renters) had higher average nitrate levels. Figure 3 shows that in the two highest Latino quartiles, proportionately more systems had average nitrate concentrations > MCL (i.e., 5% and 7% in the two higher quartiles compared with 0% in both of the lower quartiles). These two quartiles also had the largest fractions of CWSs in the medium nitrate category. The two quartiles with the lowest rates of home ownership had the largest proportions of systems in the medium and high nitrate categories (15% and 22%, respectively), compared with the two quartiles with the highest rates of home ownership (which had 7% and 8%, respectively).

Of the population served in our sample, approximately 84.6% (~ 2,494,442 people) were potentially exposed to low nitrate levels, 15.2% (~ 448,729 people) to medium nitrate levels, and 0.2% (~ 5,176 people) to high nitrate levels (Table 2). Of the 5,176 people served water with nitrates above the MCL, 56% were people of color (50% Latinos and 6% non-Latino), compared with 52% in the low and medium nitrate categories (Table 2).The percentage of Latinos served by high-nitrate CWSs was higher than the percentage of Latinos served by CWSs in the other two nitrate categories (39% and 40% for low and medium nitrate, respectively). This percentage was also greater than the percentage of Latinos in our entire study sample (39%; Table 1). This percentage was also higher than the percentage of Latinos served by CWSs in the other two nitrate categories (39% and 40% for low and medium nitrate, respectively).

**Table 2:** Demographic profile of total PEP^*a*^ in study sample by average level of nitrate concentration.

Nitrate level				
Variable of interest	Low	Medium	High
Percent total population (*n* = 2,948,346)		84.6		15.2		0.2
Percent Latino (*n* = 1,164,714)		39		40		50
Percent non-Latino people of color (*n* = 389,336)		13		12		6
Percent white (*n* = 1,394,296)		47		48		44
Low is less than one-half the MCL of 45 mg NO_3_/L; medium is one-half the MCL up to the MCL (22.5 mg/L to 44.9 mg/L NO_3_); high is equal to or greater than the MCL. ^a^Per water system, PEP is the population count of the demographic variable of interest *x* (number of point-of-entry sources in one of three nitrate levels ÷ by the total number of point-of-entry sources). PEP displayed in table is equal to the sum across all water systems. This value can also be interpreted as the estimated number of people served water at this level.						

*Model results.* Table 3 shows the multivariate modeling results. Unadjusted models indicate that percent Latino was positively and significantly [® = 0.14; 95% confidence interval (CI), 0.04–0.24] correlated with the average nitrate concentration in the distribution system. Conversely, home ownership was negatively correlated with average nitrate concentration but only marginally significant (® = –0.15; 95% CI, –0.30 to 0.003).

**Table 3:** Regression for factors associated with nitrate concentration (mg NO_3_/L) in CWSs, with beta coefficients, 95% CIs, and levels of significance.

Variable	Model A*a*	Model B*a*	Model C*b*	Model D (< 200 con.)	Model E (≥ 200 con.)
Constant		14.2 (9.1 to 19.4)^#^		27.1 (16.3 to 38.0)*		6.3 (–11.4 to 24.0)		10.8 (–32.3 to 53.9)		3.2 (–15.5 to 21.9)
Percent Latino		0.14 (0.04 to 0.24)^#^				0.04 (–0.08 to 0.16)		0.44 (0.03 to 0.84)**		–0.01 (–0.12 to 0.10)
Percent non-Latino people of color		–0.18 (–0.62 to 0.25)				–0.15 (–0.47 to 0.18)		–0.44 (–1.1 to 0.27)		–0.13 (–0.45 to 0.18)
Percent home ownership				–0.15 (–0.30 to 0.003)*		–0.16 (–0.33 to 0.002)*		–0.15 (–0.64 to 0.33)		–0.10 (–0.27 to 0.07)
Incorporated						–4.4 (–9.3 to 0.56)		–2.9 (–31.8 to 25.9)		–4.1 (–9.3 to 1.1)
Groundwater or combined*c*						9.7 (4.3 to 15.2)^#^		NA		11.7 (7.9 to 15.4)^#^
Private non-PUC regulated*d*						2.7 (–5.4 to 10.9)		5.5 (–2.7 to 13.7)		–0.23 (–4.5 to 4.1)
Public*d*						7.2 (2.8 to 11.5)^#^		10.3 (–17.4 to 38.0)		7.3 (3.6 to 11.1)
< 200 service connections						9.1 (–2.5 to 20.7)		NA		NA
Valley floor						7.9 (1.6 to 14.2)**		1.7 (–12.0 to 15.4)		7.4 (1.0 to 13.9)
2000*e*						1.3 (–0.44 to 3.1)		5.0 (1.2 to 8.8)^#^		0.71 (–1.1 to 2.6)
2001*e*						1.4 (–0.26 to 3.1)*		5.5 (1.9 to 9.1)^#^		0.67 (–0.98 to 2.3)
Summer/fall						1.3 (–0.30 to 2.9)		3.2 (0.31 to 6.3)**		1.1 (–0.72 to 3.0)
NA, not applicable, because no CWS in this model run contains this factor, or all CWSs have this factor. Data are regression statistics with robust SEs, clustered by CWSs. Coefficients represent the estimated difference in mean concentration at the system level associated with a unit change in the covariate (95% CI). Empty cells indicate that the unadjusted model included only the key variables of interest. **a**Unadjusted models, all CWSs included. **b**Adjusted model, all CWSs included. **c**Surface water is referent category. **d**Privately owned PUC-regulated CWSs are referent category. **e**1999 is referent year. *p < 0.10. **p < 0.05. ^#^*p* < 0.01.										

Our adjusted model suggests that, on average, a 1% increase in Latinos served by a CWS was associated with an increase of 0.04 mg NO_3_/L (95% CI, –0.08 to 0.16). For home ownership, each percent increase was associated with a decrease of 0.16 mg NO_3_/L (95% CI, –0.33 to 0.002). For systems with < 200 connections, the associations between percent Latino and home ownership and nitrate concentration were consistent with both the unadjusted model and adjusted model for all CWSs, but the strength of the association for percent Latino increased. Specifically, on average, each percent increase in Latino was associated with a 0.44 mg NO_3_/L increase (95% CI, 0.03–0.84) in the smaller systems. A 1% increase in home ownership was associated with a 0.15 mg NO_3_/L decrease (95% CI, –0.64 to 0.33), although the association was not statistically significant. In systems with ≥ 200 connections, neither race/ethnicity nor home ownership was associated with nitrate concentrations. Using the final model to predict expected values, we estimated that among small systems, nitrate levels for CWSs with 0% Latinos would be, on average, 6 mg NO_3_/L lower compared with CWSs at the mean.

## Discussion

To our knowledge, this is the first study to examine the relationship between nitrate levels in CWSs and social disparities in theUnited States. After stratifying by system size, we found that among systems with < 200 connections, those serving higher percentages of Latinos had higher nitrate levels. We found an inverse but not statistically significant association between home ownership and nitrate levels for smaller systems. For large systems, we did not find significant associations between race/ethnicity or home ownership and nitrate levels. Our findings corroborate previous drinking water studies (e.g., Byrne 2003) that showed a positive relationship between percent minority and poor water quality but are specific to nitrate contamination at the community level. That water quality varied by percent Latino or home ownership matters not only because of environmental equity but also because elevated nitrate levels could pose a greater hazard to subpopulations that may have less access to health care.

The association of race/ethnicity and SES with nitrate levels could be due to several factors. Race/ethnicity could have been related to proximity to agriculture, as well as the ability of residents to participate in the governance of their CWSs. For example, in systems with higher fractions of Latinos, language abilities, citizenship status, or lack of political clout could inhibit residents from speaking out and demanding improvements in water quality (for a discussion in relation to electoral politics, see Michelson 2000). Home ownership could have been negatively associated with nitrate levels because renter-based communities may have had a lower capacity to pay for improvements in water infrastructure or to hold a CWS accountable, assuming they received notices of violation as required (CCR 2008, §64463). Or, it might indicate that a lack of economic resources may influence whether CWSs can hire capable water managers or comply with regulations.

That > 5,000 people in our study sample were potentially exposed to drinking water with nitrate concentrations above the MCL raises health concerns. As noted, acute and chronic health effects have been found for vulnerable populations (e.g., infants and pregnant women) exposed to nitrate exceeding the MCL (Ward et al. 2005). Furthermore, that many of the water systems in this category had only one or two sources can lead to increased chances of high exposure for residents if these are high-nitrate sources, because there is no immediate alternative water source to draw from or blend with. These small systems often go years with high nitrate levels, until a new water source can be developed (Spath D, personal communication). PICME (CDPH 2008a) data corroborates this observation: of the 10 systems whose average nitrate concentration exceeded the MCL, 6 had recurring MCL violations over an 8-year time frame. In these systems, customers may be continually exposed (although exposure would be lower if people frequently use alternative sources). Additionally, because customers in the San Joaquin Valley often cope by purchasing bottled water, they pay two water bills—one for tap water and the other for bottled water (Moore et al. 2011).

Our results also highlight a less frequently discussed issue—approximately 448,700 residents, served by 33 CWSs, in our sample received water with medium nitrate levels (< MCL but > MCL/2). Nitrate levels in these systems could be approaching the MCL in some cases. In our study, one-third of CWSs in this category had average concentrations > 30 mg NO_3_/L. Residents in these systems could be at risk of adverse health outcomes and/or could experience additional economic costs. For example, in one study, the risk of colon cancer increased for certain susceptible subgroups (e.g., those with low vitamin C intake or high meat intake) whose water had nitrate levels > MCL/2 for at least 10 years (DeRoos et al. 2003). More generally, although several authors have suggested that the current nitrate MCL could be increased to a less stringent level of regulation (L’hirondel and L’hirondel 2002), others have argued that the standard should not be changed (Ward et al. 2005) because of uncertainties in the exposure assessment data and health effect estimates of the epidemiologic study upon which the current MCL is based (Walton 1951). Some authors also argue that the MCL includes no safety factor, and documented cases of infant methemoglobinemia have occurred at levels below the MCL (Fan and Steinberg 1996). Thus, exposure to nitrates in the middle category is important to consider.

Monitoring of water quality by these CWSs is also an important consideration. CWSs with any source whose nitrate levels exceed MCL/2 (> 22.5 mg NO_3_/L) are required to increase their monitoring frequency from annual to quarterly sampling (CCR 2008, §64432.1). At best, the cost of increased monitoring must be passed along to consumers. At worst, the funding and staffing constraints can limit the capacity of small CWSs to monitor; these CWSs may have nitrate levels approaching the MCL but neither the system operators nor customer base would know (Haberman R, personal communication). Such a scenario would undermine the aim of the SDWA, which is supposed to protect the public from harmful exposures and requires systems to notify their customers so that precautionary measures can be taken to reduce exposures (CCR 2008, §64480; Fan and Steinberg 1996).

This study used an appropriate unit of analysis (i.e., CWS) for estimating system-level nitrate exposure. The methods we used could be applied to other contaminants and to other regions of the United States. However, sources of error exist in our demographic estimate because *a*) surface intakes/well fields could fall in census block groups not served by the CWS, *b*) not all census block groups served by a CWS have an intake/field located within them, and *c*) Latinos in census data could be undercounted because of legal status. Despite these potential errors, for most CWSs, sources fell within the same census block groups that overlapped with service area boundaries of CWSs. And, for 9 of the 10 systems in the high-nitrate category, all sources were in the same census block groups as those included in each CWS service area [see Supplemental Material (http://dx.doi.org/10.1289/ehp.1002878)]. Additional sources of error include possible misclassification of points of entry to the distribution system because of errors in PICME. Furthermore, because the relative flow of different sources contributing to each CWS was not known, our method may have over- or underestimated average nitrate levels. However, at least among CWSs with average concentrations over the MCL, the estimated concentrations were similar to the measured concentrations for which that CWS received one or more MCL violations. Our measure of exposure was limited by data availability, so for systems with fewer samples tested for nitrates, our estimate may be less accurate. Although the number of persons potentially exposed to nitrate over the MCL is small, it is likely to be an underestimate of the actual population affected in the San Joaquin Valley. This is partly because our study underrepresented smaller CWSs, and partly because we used average rather than maximum nitrate levels or other measures of nitrate (e.g., MCL violations). Thus, our estimate is likely to be a conservative measure of potential exposure. The estimate of the PEP may also contain some error, because there may be some differences among utilities in how population estimates are calculated. Finally, although our results are based on data that are 10 years old, we believe that, at a minimum, they capture current trends in the San Joaquin Valley because nitrate concentrations generally change slowly in deeper public supply wells and have been increasing in most locations because of increasing fertilizer use (Dubrovsky et al. 2010).

## Conclusion

Our study is one of the first to analyze the relationships between drinking water contamination, race/ethnicity, and SES in the United States and the first that focuses on social disparities in nitrate contamination. Our results indicate that Latinos in the San Joaquin Valley may be disproportionately exposed to higher levels of nitrates and that this exposure is particularly prevalent in smaller water systems. With the increasing use of nitrogen-based fertilizers and growing demand for groundwater, these trends are likely to worsen in future years. Regulatory and policy strategies to address scale-related vulnerabilities in drinking water quality have generally ignored the environmental justice implications for CWSs. Given the U.S. EPA’s renewed focus on environmental justice (U.S. EPA 2009) and the paucity of environmental justice studies on drinking water, this study highlights the importance of targeting funding for mitigation and source water protection efforts for underserved communities and those with nitrate levels over the MCL. Furthermore, there is a need for resources to better monitor water quality and develop precautionary mitigation for communities with nitrate levels > MCL/2.

## Supplemental Material

(128 KB) PDF

## References

[r1] Burke G (2009). AP IMPACT: School Drinking Water Contains Toxins.

[r2] Byrne MS (2003). Analyzing Environmental Equity in the Public Drinking Water System [Master’s Thesis].

[r3] Calderon RL, Johnson CCJ, Craun G, Dufour AP, Karlin RJ, Sinks T (1993). Health risks from contaminated water: do class and race matter?. Toxicol Ind Health.

[r4] CCR (California Code of Regulations) (2008). Title 22, Social Security. Division 4, Environmental Health. Chapter 15, Domestic Water Quality and Monitoring Regulations.

[r5] CDPH (California Department of Public Health) (2008a). Permits, Inspections, Compliance, Monitoring and Enforcement (PICME).

[r6] CDPH (California Department of Public Health) (2008b). Water Quality Monitoring Database.

[r7] Charamandari E, Meadows N, Patel M, Johnston A, Benjamin N. (2001). Plasma nitrate concentrations in children with infectious and noninfectious diarrhea.. J Pediatr Gastroenterol Nutr.

[r8] Clean Water Act of 1972 (1972).

[r9] Committee on Small Water Systems (1997). Safe Water from Every Tap. Improving Water Service to Small Communities.

[r10] Cory DC, Rahman T (2009). Environmental justice and enforcement of the Safe Drinking Water Act: the Arizona arsenic experience.. Ecol Econ.

[r11] DeRoos A, Ward M, Lynch C, Cantor K. (2003). Nitrate in public water systems and the risk of colon and rectum cancers.. Epidemiology.

[r12] Dubrovsky NM Burow KR Clark GM Gronberg JM Hamilton PA Hitt KJ 2010 The Quality of our Nation’s Waters—Nutrients in the Nation’s Streams and Groundwater, 1992–2004. Available: http://pubs.usgs.gov/circ/1350/ [accessed 2 August 2010).

[r13] Dubrovsky NM, Kratzer CR, Brown LR, Gronberg JM, Burow KR (1998). Water Quality in the San Joaquin-Tulare Basins, California, 1992–95. U.S. Geological Survey Circular 1159.. http://pubs.usgs.gov/circ/circ1159/.

[r14] Duhigg C (2009). Millions in U.S. drink dirty water, records show. New York Times, 7 December.

[r15] Fan AM, Steinberg VE (1996). Health implications of nitrate and nitrite in drinking water: an update of methemoglobinemia occurrence and reproductive and developmental toxicity.. Regul Toxicol Pharmacol.

[r16] Glover T (2010). Westlands Water District: managing the groundwater–agriculture nexus in the largest U.S. irrigation district. In: Toward Sustainable Groundwater in Agriculture: An International Conference Linking Science and Policy, 15–17 June 2010, San Francisco, CA.. http://ag-groundwater.org/presentations/author/?uid=1174&ds=517.

[r17] Greenland S, Drescher K. (1993). Maximum likelihood estimation of the attributable fraction from logistic models.. Biometrics.

[r18] Hanukoglu A, Danon P. (1996). Endogenous methemoglobinemia associated with diarrheal diseases in infancy.. J Pediatr Gastroenterol Nutr.

[r19] Harter T. (2009). Agricultural impacts on groundwater nitrate.. Southw Hydrol.

[r20] Huber P (1967). The behavior of maximum likelihood estimates under nonstandard conditions. In: Proceedings of the Fifth Berkeley Symposium on Mathematical Statistics and Probability.

[r21] Krieger N, Williams DR, Moss NE (1997). Measuring social class in U.S. public health research: concepts, methodologies, and guidelines.. Annu Rev Public Health.

[r22] Laird NM, Ware JH (1982). Random-effects models for longitudinal data.. Biometrics.

[r23] Levin RB, Epstein PR, Harrington W, Olson E, Eric G (2002). U.S. drinking water challenges in the twenty-first century.. Environ Health Perspect.

[r24] L’hirondel J, L’hirondel J-L (2002). Nitrate and Man: Toxic, Harmless, or Beneficial?.

[r25] Lubin J, Colt J, Camann D, Davis S, Cerhan J, Severson R (2004). Epidemiologic evaluation of measurement data in the presence of detection limits.. Environ Health Perspect.

[r26] Michelson MR (2000). Political efficacy and electoral participation of Chicago Latinos.. Soc Sci Q.

[r27] Moore E, Matalon E, Balazs C, Clary J, Firestone L, De Anda S (2011). The Human Costs of Nitrate-Contaminated Drinking Water in the San Joaquin Valley.

[r28] Morello-Frosch R, Pastor M, Sadd J. (2001). Environmental justice and southern California’s “riskscape”: the distribution of air toxics exposures and health risks among diverse communities.. Urban Aff Rev.

[r29] Murphy M, Lewis L, Sabogal RI, Bell C (2009). Survey of unregulated drinking water sources on Navajo Nation [Abstract]. In: American Public Health Association 137th Annual Meeting and Exposition on Water and Public Health, 7–11 November 2009, Philadelphia, PA.. http://apha.confex.com/apha/137am/webprogram/Paper208881.html.

[r30] Oliver ML, Shapiro TM (1997). Black Wealth/White Wealth: A New Perspective on Racial Inequality.

[r31] Olmstead SM (2004). Thirsty colonias: rate regulation and the provision of water service.. Land Econ.

[r32] Pilley AK, Jacquez S, Buckingham RW, Rao SP, Sapkota K, Kumar S (2009). Prevalence of arsenic contaminated drinking water in southern New Mexico border colonias [Abstract]. In: American Public Health Association 137th Annual Meeting and Exposition on Water and Public Health, 7–11 November 2009, Philadelphia, PA.. http://apha.confex.com/apha/137am/webprogram/Paper204703.html.

[r33] Ruddy BC, Lorenz DL, Mueller DK (2006). County-Level Estimates of Nutrient Inputs to the Land Surface of the Conterminous United States, 1982–2001. U.S. Geological Survey Scientific Investigations Report 2006=5012, 17.. http://pubs.usgs.gov/sir/2006/5012/.

[r34] Safe Drinking Water Act of 1974 (1974).

[r35] Sanchez-Echaniz J, Benito-Fernández J, Mintegui-Raso S. (2001). Methemoglobinemia and the consumption of vegetables in infants.. Pediatrics.

[r36] Spalding R, Exner M. (1993). Occurrence of nitrate in groundwater—a review.. J Environ Qual.

[r37] Storm DL (1994). Chemical monitoring of California’s public drinking water sources: public exposures and health impacts. In: Water Contamination and Health (Wang RGM, ed).

[r38] U.S. Census Bureau (2000). Census of Population and Housing, 2000 [United States]: Summary Tape File 3.

[r39] U.S. Census Bureau (2007). USA Counties.. http://censtats.census.gov/usa/usa.shtml.

[r40] U.S. EPA (Environmental Protection Agency) (2009). Speeches by EPA Administrator. Administrator Lisa P. Jackson, Remarks to the National Environmental Justice Advisory Council, as Prepared.. http://yosemite.epa.gov/opa/admpress.nsf/dff15a5d01abdfb1852573590040b7f7/313ec9a2bc80d677852575fa007b3c42!OpenDocument.

[r41] U.S. EPA (Environmental Protection Agency) (2010a). Drinking Water Contaminants.. http://www.epa.gov/safewater/contaminants/index.html.

[r42] U.S. EPA (Environmental Protection Agency) (2010ba). Public Drinking Water Systems: Facts and Figures.. http://water.epa.gov/infrastructure/drinkingwater/pws/factoids.cfm.

[r43] Walton B. (1951). Survey of the literature relating to infant methemoglobinemia due to nitrate contaminated water.. Am J Public Health.

[r44] Ward MH, deKok PL, Brender J, Gulis G, Nolan B, VanDerslice J (2005). Workgroup report: drinking-water nitrate and health-recent findings and research needs.. Environ Health Perspect.

[r45] Ward MH, Kilfoy BA, Weyer PJ, Anderson KE, Folsom AR, Cerhan JR (2010). Nitrate intake and the risk of thyroid cancer and thyroid disease.. Epidemiology.

