# Supplementary material for: Social Disparities in Nitrate-Contaminated Drinking Water in California’s San Joaquin Valley
Source: Environ Health Perspect. 2011 Jun 3;119(9):1272–8. doi: 10.1289/ehp.1002878 (PMC3230390; doi:10.1289/ehp.1002878)
Supplement: (128 KB) PDF [file ehp.1002878.s001.pdf]

Supplemental material:

Title: Social Disparities in Nitrate Contaminated Drinking Water in California's San Joaquin Valley

Authors: Carolina Balazs, Rachel Morello-Frosch, Alan Hubbard, Isha Ray

Table of Contents

Estimating customer demographics

- Digitized boundaries and aerial weighting approaches
- Surface intake/well field-based approach
- Assumptions and sources of error
- Goodness-of-fit test

Characteristics of CWS with average nitrate over the MCL

Abbreviations

CWS – Community water system

GIS – Geographic Information System

MCL – Maximum contaminant level

PICME – Permits, Inspections, Compliance, Monitoring and Evaluation database

Estimating customer demographics

Digitized boundaries and aerial-weighting approach

We estimated the demographics of community water systems (CWS), our units of interest for this study, given data availability. We employed two methods to estimate these demographics, and selected one for final use. In the first method, we collected hard and digital copies of system boundaries and digitized these in GIS for two pilot counties—Fresno and Tulare. We then estimated water system demographics by using digitized water system boundaries for all CWS in Fresno and Tulare counties and spatially joining these boundaries to block groups in GIS. We used the resulting area of block groups falling within the service area to create an aerial-based weight for the demographics. The formula is:

[1]

$$Z_i = (\sum_{j=1}^{j=n}[(x_j / X_j) * p_j] / \sum_{j=1}^{j=n}[(x_j / X_j) * P_j]) \times 100$$

Where Z is the percent of the variable of interest (i.e. percent Latino) in system *i; j* identifies a particular Census block group*;* p_j is the population count of the variable of interest (e.g. white, Latino, number of owner-occupied units, etc) in Census block group j; x_j refers to the area of the Census block group *j* overlapping with the water system boundaries; X_j refers to the total area of Census block group; P_j refers to the total population in Census block group j. The numerator is the aerially weighted sum of the variable of interest, whereas the denominator is the population weighted total. While aerial weighting is widely used when estimating demographic statistics in GIS, it assumes

that the population within the Census block (or block group) is homogenously distributed.

Surface intake/well field-based approach

We compared the aforementioned approach to a second estimation procedure, which we ultimately used. This second approach is a population-weighted average that joined surface intake and well field locations ("intakes/fields", which we also refer to as "sources") to block groups, but did not weight aerially. The formula is:

[2]

$$Z_i = \sum_{j=1}^{n}(p_j + ...p_n) / \sum_{j=1}^{n}[(P_j + ..P_n]$$

Z_i refers to the percent of the variable of interest in system *i*, p_j refers to the population count of the variable of interest in Census block group j (e.g. number of Latinos), in which a given well field/intake falls; and P_j refers to the total population in block group j.

Assumptions and Sources of Error

Because no demographic information exists for CWS in the Valley, state or nation, both approaches described are estimates of reality. Each contains several sources of error, making demographic estimates from either imperfect, though reasonable, given data limitations. Sources of error in the boundary-digitized approach (approach 1) can derive from: 1) inaccurate boundaries and 2) assumptions of homogeneity of the population. Sources of error using source locations joined to block groups (approach 2) can derive from: 1) Well fields/intake locations falling outside the CWS service area, or at least the

block group served by the CWS, 2) exclusion of block groups that did not have a well field/intake location.

Conducting a detailed quantification of the error from the digitized boundary approach is beyond the scope of the paper. However, we quantified potential *sources of error* by seeing how often the aforementioned situations arose using the well field/intake location-based approach in Tulare and Fresno counties (our pilot counties). Among the 249 systems in these two counties (for which we had digitized boundaries), we found that 93% of systems had all of their intakes/fields within at least one block group that is served by some portion of the CWS (this may mean that not all block groups served by the CWS had a source in them). We found that 491 block groups (among 106 CWS) do not have an intake/field located within them. This represents a significant fraction of the total systems assessed (42%), and likely explains some of the difference when comparing our aerial weighting method to our intake/field method (suggesting we would encounter similar error in our study sample). However, without knowing the true demographic of each system, it is difficult to say how much this impacts the demographic estimate.

For our study sample, for eight of the ten systems whose average was over the MCL, all sources were within the CWS service area and shared the same block groups as those served by the CWS. The ninth system had all sources within the same block group as that served by the CWS, and the sources were within 500 feet of the community. The tenth had two-thirds of its sources in block group not served by the CWS. Thus, while

there may be some error due to our use of block group estimates, we expect minimal error for these systems.

Goodness-of-fit test

In addition to spatial comparisons, we compared our two approaches—digitized boundary with aerial weighting (approach 1) to sources joined to block groups (approach 2) by running a goodness-of-fit test. This test regressed estimates of percent Latino and percent homeownership from the latter approach against demographic estimates from the former approach. This allowed us to assess how close both methods were to each other. By examining the R^2 values for our two key variables of interest (percent Latino and percent home ownership) we determined our source-based approach reasonably (i.e. $R^2>=.80$) resembled the digitized approach, especially for the percent Latino estimate. The R^2 is lower for home ownership ($R^2=.48$). Given this comparison, the fact that neither approach is the "gold standard", and the fact that digitized boundaries were not available across the Valley, we concluded that using source locations was a reasonable approach.

Characteristics of CWS With Average Nitrate Over the MCL

Supplemental Material, Table 1. Description of 10 systems in study sample with average nitrate concentrations above the MCL.

System	Ownership Type	# of Sources with samples (proxy for # of sources)	Estimated average nitrate concentration[a] (mg NO_3/L)	Years for which MCL violations issued and associated nitrate concentration (PICME 2008) [b]	Difference[c] between estimated average nitrate concentration and reported concentration in year for which MCL violation was given (mg NO_3/L)
1	City Tract	2	48.7		No violation in time period[d]
2	Private, Mutual	1	69	2001 (69)	0
3	Private, mutual	1	66	2000 (110)	-44
4	Irrigation District	6	56.7	1999 (57)<br>2000 (67)	-1.7 in 1999<br>-10.3 in 2000
5	Private, Labor Center	2	56.1	2000 (73)<br>2001 (78)	-16.9 in 2000<br>-21.9 in 2001
6	Private, Labor Camp	1	150	2000 (150)	0
7	Private, Mutual	2	47.6	1999 (80)	-32.4
8	Private, Mutual	1	51.3	2000 (48)	-3.3
9	Private, Mutual	3	62.8	2000 (47.9)<br>2001 (54)	14.9 in 2000<br>8.8 in 2001
10	Private, Labor Center	1	104.4	1999 (115)<br>2000 (106)<br>2001 (96.75)	-10.6 in 1999<br>-1.6 in 2000<br>7.65 in 2001

[a] Estimated average nitrate concentration derived from study.
[b]Data source for year of violation and concentration of violation (at the source-level) derived is the Permits, Inspections, Compliance, Monitoring and Evaluation (PICME) database. Nitrate concentration in mg NO_3/L.
[c]Where a system had more than one year with a violation, the difference is noted for each year. A negative number denotes that system-level average was below the concentration for which the MCL was given.
[d]MCL violation in 1998 for 46.5 mg NO_3/L, just one year before study period.

References

Permits Inspections Compliance Monitoring and Enforcement (PICME). 2008. California Department of Public Health. Sacramento, CA.
